# Risk factors for prevalent hepatitis C virus-infection among inmates in a state prison system in Mexico

**DOI:** 10.1371/journal.pone.0179931

**Published:** 2017-06-27

**Authors:** Pablo F. Belaunzarán-Zamudio, Juan L. Mosqueda-Gomez, Alejando Macias-Hernandez, Juan G. Sierra-Madero, Saifuddin Ahmed, Chris Beyrer

**Affiliations:** 1 Department of International Health, Johns Hopkins Bloomberg School of Public Health, Baltimore, Maryland, United States of America; 2 Departamento de Infectología, Instituto Nacional de Ciencias Médicas y Nutricion Salvador Zubiran, Mexico City, Mexico; 3 División de Investigación de la Facultad de Medicina, Universidad Nacional Autónoma de México, Mexico City, Mexico; 4 Departamento de Microbiología, Universidad de Guanajuato, León, Guanajuato, Mexico; 5 Centro Ambulatorio para la Prevención y Atención en SIDA e Infecciones de Transmisión Sexual (CAPASITS), León, Guanajuato, Mexico; 6 Department of Epidemiology, Johns Hopkins Bloomberg School of Public Health, Baltimore, Maryland, United States of America; 7 Johns Hopkins Center for Public Health & Human Rights, Johns Hopkins Bloomberg School of Public Health, Baltimore, Maryland, United States of America; British Columbia Centre for Excellence in HIV/AIDS, CANADA

## Abstract

**Objectives:**

To estimate the prevalence of HCV-infection and identify associated factors among inmates in the State Prison System of Guanajuato in Mexico (Sep-2011 to Feb-2012).

**Methods:**

Cross-sectional, observational study in 10 prisons in the State of Guanajuato in Mexico (2011–2012). We offered HCV-testing and applied audio computer-assisted self-interviews to all adults imprisoned in the State Prison System. We used a complex survey analysis to estimate the distribution of variables and its corresponding 95% confidence intervals, taking into consideration the expected cluster effect by common characteristics within prisons. Inverse probability weights were applied to correct potential biased estimates arising from non-participation in accrual activities and non-response rates. We fitted multivariate logistic regression models to identify risk-behaviors associated to HCV-infection.

**Results:**

We included data of 2,519 participating inmates. Prevalence of HCV-infection was 4.9 (95%CI = 3.6–5.9). Most HCV-infected inmates were male (99%). Before being incarcerated, inmates with HCV-infection were more frequently tattooed, used and injected drugs more frequently, and were more likely to share materials for injecting, when compared with those non-infected. During incarceration, HCV-infected inmates got tattoos and used drugs more often than non-infected, including injecting-drugs and sharing materials. Injecting-drug use (OR = 7.6, 95%CI, 2.5–23.4), sharing materials for injecting-drugs (OR = 19.6, 95%CI, 4.7–81.7) and being tattooed at least once before incarceration (OR = 2.1, 95%CI, 1.1–3.9), but not during incarceration, were independently associated to HCV-infection.

**Conclusions:**

The prevalence of HCV-infection among inmates in the State of Guanajuato in Mexico is considerably higher than in the general population. The most important risk factors for HCV in this inmate population were injecting-drugs and sharing materials for injections before incarceration. High-risk behaviors during imprisonment are very high particularly among those already infected. HCV diagnostic and treatment services, and harm-reduction programs for incarcerated injecting-drug users in Mexico should be integrated to control the HCV epidemic in Mexico.

## Introduction

Prison inmates have a higher prevalence of Hepatitis C Virus (HCV) than the general population [1,2]. They are a highly-selected population among those at high-risk [3,4]. In addition, the restrictions in access to preventive health services in prisons increases the frequency of high-risk behaviors and exposure [5,6]. The HCV-infection epidemic in prisons is closely related to injecting-drug use (IDU) and the high rate of incarceration among people who inject drugs (PWIDs) [1,2,4,5]. While tattooing has been characterized as a risk-behavior associated with IDU, there is also evidence that tattooing is associated to HCV-infection, particularly among inmates. Several studies have shown that among injecting and non-injecting incarcerated drug users, tattooing is independently associated to HCV-infection [1,6,7]. Also, prisoners who acquired their tattoo in prisons are more likely to be HCV-infected [8].

Mexico holds one of the largest populations of prisoners in the world [9], but little is known about the HCV epidemic in this population [10,11]. In countries with highly punitive policies against drug use, high prevalence of drug use or both, the prison population is drawn heavily from the PWIDs [12–14]. The role of IDU among inmates in the prevalence of HCV infection in settings with low frequency of IDU however, is less studied. In this report, we explored the association between demographic characteristics, individual drug-use and tattoo practices, and HCV infection among inmates in the State Prison System of Guanajuato, Mexico during 2011–2012. Using a survey and blood tests data, we tested the hypothesis that in this group of inmates amongst whom IDU would likely have a low prevalence, having a tattoo within the prison would be independently associated to HCV infection.

## Materials and methods

This observational cross-sectional study was nested within an HIV testing program that the General Directorate for Penal Execution and Social Re-Adaptation (DGEPRS) of the Ministry of Public Safety in the State of Guanajuato in Mexico implemented in 2011–2012 with support of the State’s Ministry of Health (MOH). The DGERPS manages 10 Centers for Social Re-insertion (CERESOs), each one located in a different city within the State. The centers held a constantly changing population of convicted and non-convicted inmates of both sexes that varied between 4,500–5,500 through the year. The population size in each center varied between 100 and 1,500 inmates and the largest 2 centers account for about 50% of the total population.

All adults imprisoned in the 10 CERESOs between August 2011 and February 2012 were eligible for participation. Data was collected using two different procedures: blood tests results and application of structured questionnaires. Inmates had the option of participating in blood tests, answering the questionnaire or both.

All inmates were offered counseling and testing, regardless of their participation in the research study. After the counseling and testing session, we invited them to voluntarily participate in the research study through a written informed consent process. Inmates had the option to be tested without participating in the research study. A separate consent for anonymously using their blood tests results was requested. They were also offered the option to participate in the study either consenting to the use of their blood test results, answering the survey, or both. Inmates who could not give their informed consent due to language barriers, mental health or neurologic conditions were excluded.

Blood samples were drawn and coded using a random numbers sequence, labeled, centrifuged and transported to the State Center for Blood Transfusion of the State of Guanajuato (Centro Estatal de la Transfusión Sanguínea–CETS-), where diagnostic tests were performed. Detection of plasma specific antibodies against HCV (AbHCV) were performed using a 4th generation assay (ARCHITECT i2000 system, Abbott Diagnostics, Wiesbaden-Delkenheim, Germany). HCV-infections were confirmed with a Cobas AmpliPrep/Cobas TaqMan HCV quantitative test, version 2.0 (CAP/CTM HCV test, v2.0) assay. HCV-infected inmates were referred to the closest MOH’s hospital to receive healthcare. Structured questionnaires contained questions about demographic characteristics, information related to the current incarceration, and individual risk behavior before and during incarceration. We used an Audio-assisted Computer Administered Self-Interview (A-CASI) system for questionnaires (participants were assisted by study staffs when requested). The numerical code for blood tests was used to identify questionnaire respondents and link survey data to blood tests results.

HCV-infection was coded as a binary variable according to the laboratory results. All other variables were defined as self-reported by participants in the survey. Inmates were asked whether they had ever been tattooed before and during the current imprisonment; and whether they had ever used drugs before and during imprisonment. Inmates, who admitted using drugs during any of the periods inquired, were specifically asked about IDU during that period. To assess the frequency of material sharing for injecting in the CERESOs, inmates were asked whether they knew if any of the materials used for injecting had been previously used by other people to inject. All questions could be answered as “Yes”, “No” or “Prefer not to answer”.

Before the study, we assumed that prevalence of IDU and tattoo were 1% and 50%, respectively, and that the true prevalence of HCV-infection was 5%. Expecting a refusal rate of 25% among 5,500 inmates, we estimated a low power to identify associations with a low frequency risk behavior, such as IDU (66% for RR = 5) but adequate power to identify risk associated to tattoo (96% for RR = 2). We used a complex survey analysis to estimate the distribution of variables of interest with its corresponding 95% confidence intervals taking into consideration the expected cluster effect by common characteristics within CERESOs. We used inverse probability weights to correct potential biased estimates arising from non-participation in accrual activities and non-response rates, by gender and CERESO. The Taylor linearization method was used to estimate standard errors of weighted proportions. Missing data on individual risk behaviors was imputed using multiple imputations through a multivariate imputation via chained equations (mice) [15]. Data analysis was performed using STATA version 11 [16]. To test the hypothesis that in this group of inmates with expected low frequency of IDU, having a tattoo in the prison would be the most important behavioral risk associated to HCV infection, [10,11] we fitted a pre-specified multivariate logistic regression model that included variables previously associated to HCV-infection.

The study was reviewed and approved by the Johns Hopkins Bloomberg School of Public Health Institutional Review Board, and the Bioethics Committees of the MOH and the University of Guanajuato. We abided to the principles of the Declaration of Helsinki and the Belmont Report.

## Results

### Description of participants and prevalence of HCV-infection by demographic, imprisonment and behavioral characteristics

We included data on 2,519 inmates that consented to participate in both, blood tests result and available survey data, even if they did not complete the entire survey. There were 123 people with HCV infection, for an overall crude prevalence of HCV-infection of 4.9% [17]. We summarized the characteristics of the study population and the prevalence of HCV infection by demographic, incarceration and behavioral characteristics in Table 1. Briefly, the study population consisted mostly of young (mean age 34yo), male inmates (95%). Before being imprisoned, 58% of participants were married or cohabiting with their partners. A high proportion (30%) had been previously imprisoned (half of them more than once), and had been imprisoned a median of 3 years (p25-p75, 2–6 years), the day of the study (See Table 1).

**Table 1:** Demographic characteristics of inmates in the State Prison System in Guanajuato, Mexico (Sep 2011-Feb 2012) (*N* = 2,519)^a^.

	All participants		Prevalence of HCV-infection	
Demographic characteristics ^*b*^	N	(%) ^*c*^	N	%[95%CI]^*d*^
**Gender** (n = 2,519)				
Female	163	(5.4)	2	1.2[0–2.7]
Male	2,356	(94.6)	121	5.1[4.0–6.3]
**Age categories** (n = 2,479)				
18–27 years	738	(30.0)	10	1.3 [0.5–2.2]
28–37	962	(38.7)	64	6.7 [5.2–8.2]
38–47	517	(20.6)	40	7.9 [4.3–11.5]
48–57	186	(7.3)	5	2.6 [0.8–4.3]
>58	76	(3.3)	4	4.7 [0.6–8.8]
**Marital status** (n = 2,279)				
Single	739	(31.8)	42	5.8 [3.2–8.4]
Married/Co-habiting	1,321	(58.5)	65	5.0 [3.4–6.6]
Divorced/Separated	173	(7.7)	5	2.7 [0.1–4.5]
Widowed	35	(1.5)	1	2.6 [0–7.5]
Other	11	(0.5)	0	0
**Employment** ^*e*^ n (%) (n = 2,114)				
Yes	1,930	(90)	89	4.9 [3.7–6.0]
No	150	(8.2)	11	6.4 [2.5–10.4]
Housewife	34	(1.8)	0	0
**Education** ^*f*^ n (%) (n = 2,103)				
Less than 9 years	1,113	(53.0)	64	6.0 [4.5–7.4]
9 years or more	990	(47.0)	35	3.7 [2.1–5.3]
**Captive in municipal jail right before being transferred to current center** (n = 1,998)				
No	1,168	(58.5)	43	3.7 [2.6–4.9]
Yes	829	(41.5)	55	6.7 [5.1–8.3]
**Previously incarcerated** (n = 1,942)				
No	1,413	(71.1)	35	3.1 [1.8–4.5]
1 time	257	(13.9)	9	4.6 [0–9.4]
≥2 times	272	(15)	43	13.7 [10.8–16.5]
**Length of incarceration** (n = 1,874)				
< 1 year	841	(45.7)	32	3.8 [2.3–5.2]
1–2 years	260	(13.9)	23	8.8 [7.0–10.6]
3–5 years	429	(22.4)	20	4.7 [2.0–7.4]
6–10 years	247	(12.8)	9	3.6 [1.0–5.8]
>10 years (Range: 11–56)	97	(5.1)	7	7.2 [2.4–11.6]
	**Before incarceration**			
**Tattoo use** (n = 1,919)				
No tattoos	1,100	(55.7)	20	2.6 [1.7–3.5]
1 tattoo	181	(9.9)	6	4.3[1.5–7.1]
2–4 tattoos	352	(18.8)	25	7.1[4.1-10-1]
>4 tattoos	288	(15.6)	32	10.8[7.3–14.3]
**Tattoo in a previous imprisonment** (n = 969)				
No	809	(65.7)	60	4.9[3.1–6.8]
Yes	104	(14.3)	18	8.5[3.3–13.6]
Don’t remember	56	(20.0)	1	2.5[0–5.3]
**Drug use** (n = 2,032)				
No	766	(37.5)	11	2.3[0.8–3.8]
Drug use, but no injecting	1,073	(53.0)	18	2.7[1.8–3.5]
Injecting drug use, no sharing	101	(5.0)	22	19.1[9.8–28.5]
Shared materials for injecting drugs	84	(4.0)	40	40.3[25.9–54.6]
Injecting drug use, don’t know if shared	8	(0.4)	1	14.1[0–35.3]
**Men having sex with men** (n = 1,784)				
No	1,547	(85.3)	71	4.9 [3.7–6.1]
Yes	237	(14.7)	20	9.6[6.7–12.5]
	**During incarceration**			
**Tattoo use** (n = 1,914)				
No tattoos	1,517	(78.4)	65	4.4[3.3-5-5]
1 tattoo	144	(7.5)	6	4.9[0.2–9.5]
2–4 tattoos	154	(8.4)	13	7.6[3.4–11.8]
>4 tattoos	99	(5.6)	10	7.7[2.1–13.3]
**Shared materials for tattooing** (n = 412)				
No	203	(49)	17	8.4[5.0–11.9]
Yes	128	(31)	10	7.7[3.4–11.9]
I don’t know	81	(19)	3	3.7[0–8.1]
**Drug use** (n = 2,005)				
No	1,339	(66)	46	3.7[2.7-4-7]
Drug use, but no injecting	628	(32)	38	6.1[3.9–8.4]
Injecting drug, not sharing	22	(1.1)	4	15[0–35.8]
Shared materials for injecting drugs	6	(0.6)	6	45.5[9.2–81.7]
Injecting drug use, don’t know if shared	5	(0.3)	2	34.2[0–83.3]
**Men having sex with men** (n = 1,024)				
No	961	(92.3)	54	5.2[3.7–6.8]
Yes	63	(7.7)	7	9.5[1.9–17.1]

^a^ Participants were included in this analysis if their blood tests result and survey data, were available and they had consented to share this information for the study, even if they did not complete the entire survey.

^*b*^ Missing data was imputed using multiple chain equation multivariate models for multiple imputations.

^*c*^ Weighted proportions according to the inverse probability of refusal participation by gender and center.

^*d*^ Employment status before imprisonment. Housewife option was not available for male inmates

^*e*^ 95% Confidence Intervals estimated using the Taylor linearization method with stratification by gender and cluster effect by center.

^*f*^ National median: 9 years

The prevalence of HCV was higher among male participants compared to female participants (5.1 vs. 1.2%). Inmates aged between 28–47 years old, those reporting to be single or unemployed before incarceration, and those with less than 9 years of education had higher HCV-prevalence of infection than their counterparts (Table 1). The distribution of the prevalence of HCV-infection was associated to the frequency of previous imprisonments and to the patterns of their risk-behaviours. For instance, we observed an increasing gradient in the prevalence of HCV-infection as the frequency of previous imprisonments increased, and as the number of tattoos before imprisonment increased (Table 1). Also, the highest prevalence of HCV-infection occurred among people that injected drugs before imprisonment (40%, 95%CI 25.9–54.6) and during the current imprisonment (45%, 95%CI, 9.2–81.7%) (Table 1).

### Individual risk behaviors associated to HCV-infection

In univariate logistic regression analyses, we observed that sex, increasing age, increasing number of previous imprisonments, increasing number of tattoos before imprisonment, injecting drugs use and sharing materials for injecting drugs before imprisonment were all associated with a statistically significant increased risk of HCV-infection (Table 2). Among male inmates, having had sex with men before imprisonment was also associated to an increased risk of infection (Table 2). In contrast, the occurrence of these same risk-behaviors during imprisonment was either not associated to HCV-infection (number of tattoos) or had a weaker association, as measured by the magnitude of the association, as was the case of injecting drug use and sharing materials for injecting drugs. We describe in detail the associations between these variables and HCV-infection in Table 2.

**Table 2:** Individual variables associated to HCV infection among adult inmates in the State Prison System of Guanajuato, Mexico (Aug 2011-Feb 2012) (n = 2,519)^a^.

Individual characteristics and risk behaviors	Univariate model			
	OR^*b*^	(95%CI) ^*b*^	*p-*value^*c*^	
**Gender**				
Male vs. Female	**4.3**	**(1.2–15.1)**	**0.023**	
**Age group (in years)**				
18–27	1.0	Reference		
28–37	**5.4**	**(3.2–9.2)**	**<0.001**	
38–47	**6.4**	**(2.7–15.7)**	**<0.001**	
48–57	2.0	(0.8–5.0)	0.138	
>58	**3.7**	**(1.1–13.1)**	**0.044**	
**Previously incarcerated**				
No	1.0	Reference		
1 time	1.4	(0.6–3.2)	0.479	
>1 time	**5.0**	**(3.2–7.6)**	**<0.001**	
**Tattoo before incarceration**				
No	1.0	Reference		
1 tattoo	1.5	(0.6–3.5)	0.077	
2–4 tattoos	**2.7**	**(1.6–4.5)**	**0.008**	
>4 tattoos	**4.1**	**(2.5–6.7)**	**<0.001**	
**Drug use before incarceration**				
No	1.0	Reference		
Drug use, but no injecting	1.1	(0.5–2.4)	0.663	
Injecting drug use, not sharing	**9.9**	**(4.4–22)**	**<0.001**	
Shared materials for injecting drugs	**30**	**(9.1–99)**	**<0.001**	
Injecting drug use, don’t know if shared	**4.5**	**(0.8–27)**	**0.030**	
**Men having sex with men before incarceration**, (Yes *vs*. No)	1.65	(0.9–2.9)	0.075	
**Captive in municipal jail right before being transferred to this center**, (Yes *vs*. No)	**1.8**	**(1.2–2.5)**	**0.004**	
**Tattoo during incarceration**				
No	1.0	Reference		
Once	1.1	(0.4–2.9)	0.832	
2–4 times	**1.8**	**(0.9–3.6)**	**0.077**	
5 times	1.9	(0.9–4.3)	0.136	
**Drug use during incarceration**				
No	1.0	Reference		
Drug use, but no injecting	**1.7**	**(1.0–2.8)**	**0.043**	
Injecting drug use, not sharing	**4.5**	**(1.1–17.5)**	**0.050**	
Shared materials for injecting drugs	**21.6**	**(3.9–118.9)**	**0.002**	
Injecting drug use, don’t know if shared	**13.4**	**(1.4–125.2)**	**0.026**	
**Men having sex with men during incarceration**, (Yes *vs*. No)	2.0	(0.7–5.7)	0.137	

^a^ Missing data was imputed using multiple imputation by chained equations procedure (mice) to estimate the proportions and 95%CI with 50 imputed datasets.

^b^ 95% Confidence Intervals estimated using the Taylor linearization method with stratification by gender and cluster effect by center.

^c^ *t-*test estimated from univariate logistic regression models

Using multivariable logistic regression models to control for the potential effect of confounders, we observed a statistically significant tendency towards increasing odds of HCV-infection by increasing age group, and number of tattoos before incarceration but not of number of tattoos during incarceration (Table 3). Drug use before incarceration was also associated to HCV-infection: people who injected drugs (OR 9.9, 95% CI = 4.4–22) and shared materials for IDU before incarceration (OR 30, 95% CI = 9.1–99) were more likely to be HCV-infected when compared to those who never used drugs. In contrast, people that injected during imprisonment (OR = 4.3, 95%CI = 1.1–17.5) and that shared materials for injecting (OR = 30, 95%CI9.1–9.9) during imprisonment were at higher odds of being infected by HCV than those not using drugs, but these associations were not observed after adjusting for all other variables (Tables 2 and 3).

**Table 3:** Multivariate analysis of individual variables associated to HCV infection among adult inmates in the State Prison System of Guanajuato, Mexico (Aug 2011-Feb 2012) (n = 2,519)^a^.

Individual characteristics and risk behaviors	Multivariate model		
	OR^*b*^	(95%CI)^*b*^	*p-*value^*c*^
**Gender**			
Male vs. Female	2.2	(0.6–8.5)	0.228
**Age group (in years)**			
18–27	1.0	Reference	
28–37	**5.1**	**(2.8–9.2)**	**<0.001**
38–47	**8.2**	**(3.3–20.2)**	**<0.001**
48–57	**3.6**	**(1.3–9.7)**	**0.016**
>58	**11.2**	**(2.9–43.2)**	**0.002**
**Previously incarcerated**			
No	1.0	Reference	
1 time	1.1	(0.4–8.7)	0.872
>1 time	2.0	(0.9–4.4)	0.068
**Tattoo before incarceration**			
No	1.0	Reference	
1 tattoo	**2.1**	**(1.1–3.9)**	**0.023**
2–4 tattoos	**2.4**	**(1.2–4.6)**	**0.021**
>4 tattoos	1.9	(0.9–4.0)	0.065
**Drug use before incarceration**			
No	1.0	Reference	
Drug use, but no injecting	**1.0**	**(0.4–2.5)**	**0.912**
Injecting drug use, not sharing	**7.6**	**(2.5–23.4)**	**0.002**
Shared materials for injecting drugs	**19.6**	**(4.7–81.7)**	**0.001**
Injecting drug use, don’t know if shared	4.8	(0.5–45.0)	0.145
**Captive in municipal jail right before being transferred to this center**, (Yes *vs*. No)	1.2	(0.80–1.77)	0.361
**Tattoo during incarceration**			
No	1.0	Reference	
Once	0.9	(0.3–3.0)	0.861
2–4 times	0.9	(0.4–2.3)	0.921
5 times	0.8	(0.2–2.7)	0.611
**Drug use during incarceration**			
No	1.0	Reference	
Drug use, but no injecting	0.8	(0.0–1.3)	0.259
Injecting drug use, not sharing	0.9	(0.1–6.7)	0.878
Shared materials for injecting drugs	1.5	(0.3–8.1)	0.589
Injecting drug use, don’t know if shared	0.8	(0.1–4.7)	0.757

^a^ Missing data was imputed using multiple imputation by chained equations procedure (mice) to estimate the proportions and 95%CI with 50 imputed datasets.

^b^ 95% Confidence Intervals estimated using the Taylor linearization method with stratification by gender and cluster effect by center.

^c^ *t-*test estimated from multivariate logistic regression models

## Discussion

In this observational study, we describe the prevalence of drug-use and tattoo practices and its association to HCV-infection in the 10 Centers for Social Re-Insertion (CERESO) of the State of Guanajuato in Mexico during 2011 and 2012. We found that inmates with HCV-infection distinctively had frequent high-risk behaviors before and during incarceration, this is consistent with previously observed behaviors for inmates with HCV-infection in different settings. Consequently, we observed a higher prevalence of HCV-infection among inmates that were tattooed before the current incarceration, and particularly among those whose tattoos had been applied in previous incarcerations. Also, the prevalence of HCV-infection was overwhelmingly higher among inmates who injected drugs and shared materials for injecting drugs before and during imprisonment. Nonetheless, after adjusting for the potential confounding effect of sex, age, and other high-risk practices, only injecting drugs and sharing materials for injecting drugs before incarceration were significantly associated to an increased risk of HCV-infection. While we hypothesized that in a population with low frequency of IDU and HCV-infection, having a tattoo within the prison would be an important associated risk factor for HCV-infection, we actually observed that the number of tattoos received during current imprisonment was not associated to a significantly increased risk of HCV-infection neither in the unadjusted analysis, nor adjusting for potential several confounding factors.

Our findings are at odds with previous reports that have clearly identified application of tattoos in prisons as an associated factor to HCV-infection, particularly among women and drug injectors, [8, 18, 19] but in agreement with a recent meta-analysis including 30 studies among prison inmates [20]. In the latter study, the heterogeneity of HCV seroprevalence among the different studies was largely explained by differences in the frequency of IDU and differences in HCV seroprevalence among IDU. One limitation of this meta-analysis is that both tattooing before and during incarceration were pooled, and no distinction was made between outside and inside prison behavior. Our study contributes to improve the knowledge about the HCV-epidemic among PWID and prison inmates by providing evidence that, despite the high frequency of high-risk tattoo practices within prisons, IDU and high-risk injecting practices play a more important role in the transmission of HCV-infection among inmates; despite the low prevalence of IDU, possibly due to the overall low prevalence of HCV.

In contrast, IDU was the most strongly associated behavior to HCV-infection. While reported use of injecting-drugs was high for the local context, it is low in comparison to studies in prisons elsewhere [1,2,5]. Nonetheless, in a country like Mexico where 5.5% of people between 12 and 65 years has used drugs during their lifetime and less than 0.1% have ever injected drugs, the frequency of drug use and injecting drug use among inmates can be considered a public health and human rights emergency by itself [21]. Our findings suggest that in Mexico, as anywhere else worldwide, there is an ongoing incarceration epidemic among people who inject drugs (PWIDs) [1,2,4,5]. The relevance of these findings and its implications for the implementation of HCV and other blood-borne infections preventive programs should not be understated, as high-risk practices for IDU is a leading factor associated with HIV and HCV among inmates worldwide [22–24].

The proportion of inmates that reported receiving tattoos with previously used materials or that did not know, was very high (50%), as was the proportion of people who injected during imprisonment that shared materials for injecting drugs or did not know whether materials for injecting had been used previously by other inmates (50%). These circumstances are related to the prison-environment determined by specific physical spaces, prisons policies and social norms within prisons; all of them independent of individual inmates. These finding strongly support that these state prisons and local jails are high-risk environments for HCV, despite the lack of association between HCV-infection and high-risk tattoo and injecting drug use practices in this study [25]. Prisons in Russia, Thailand and Canada have been previously identified as risk-environments for HIV among IDU [6,18,22]. Moreover, the venue where tattooing occurs appears not to pose an increased risk for HCV except where tattoos are applied in prison settings or by friends [26]. In this setting, it is important to highlight that structural interventions to reduce or stop sharing and reuse of equipment for tattoo, IDU and other forms of skin penetration, as recommended by UNAIDS/UNODC [27] is worth considering. Although legalization of tattoos in prisons and access to sterile materials for tattoos have been contentious in the past, [28,29] the magnitude of the problem and the potential threats of parenteral transmission of HBV, HCV or HIV requires reconsidering the policy of prohibiting tattoos and denying access to harm-reduction services in prisons.

We acknowledge that this cross-sectional study is particularly susceptible to selection bias, [30] and that obtaining reliable data on sensitive behaviours remains challenging [31]. Moreover, selection bias is particularly concerning for us considering the high proportion of refusal to participate, and dissimilar characteristics between participants and non-participants. Notwithstanding these limitations, our results are overall consistent to what has been found previously among prison inmates. Moreover, the use of narrowly defined and clearly selected population, a priori definition of variables, the use of self-applied structured questionnaire, and the systematic implementation of study procedures limit the role of bias [30]. In addition, the use of self-applied computerized interviews appears to significantly reduce reporting biases, can improve data quality [32] and can be an accurate and reliable method to collect sensitive data [33–35]. We also used standard and robust analysis methods to attempt to correct biases arising from non-participation and missing data.

In conclusion, we observed despite the low prevalence of IDU and HCV-infection in the general adult Mexican population, inmates in this prison system in Mexico are at increased risk for HCV, and possibly other blood-borne infections. This increased risk is associated with a high frequency of high-risk behaviors before and during imprisonment; and apparently by the lack of access to proper care for substance use and harm-reduction services. Injecting drug use before incarceration appears to be the single most important factor leading the HCV-epidemic in this setting. Although the prevalence of HCV is relatively low in comparison to what has been found in other prisons, a carefully planned but prompt response including interventions to reduce or halt the use of shared or recycled materials for tattooing and injecting drugs, and providing treatment for drug abuse and addiction for inmates in need of it, are urgently needed.

## Supporting information

S1 File dataset_hcv_161229.txt. (TXT)
